# Radial nerve compression: anatomical perspective and clinical consequences

**DOI:** 10.1007/s10143-023-01944-2

**Published:** 2023-02-13

**Authors:** Andrzej Węgiel, Piotr Karauda, Nicol Zielinska, R. Shane Tubbs, Łukasz Olewnik

**Affiliations:** 1 Department of Anatomical Dissection and Donation, Medical University of Lodz, Lodz, Poland; 2 Department of Anatomical Sciences, St. George’s University, Grenada, USA; 3 Department of Neurosurgery, Tulane University School of Medicine, New Orleans, LA USA; 4 Department of Neurology, Tulane University School of Medicine, New Orleans, LA USA; 5 Department of Structural and Cellular Biology, Tulane University School of Medicine, New Orleans, LA USA; 6 Department of Surgery, Tulane University School of Medicine, New Orleans, LA USA; 7 Department of Neurosurgery, Ochsner Medical Center, New Orleans, LA USA

**Keywords:** Radial nerve compression, Entrapment, Arcade of Frohse, Radial tunnel syndrome, Posterior interosseus nerve syndrome, Wartenberg syndrome

## Abstract

The radial nerve is the biggest branch of the posterior cord of the brachial plexus and one of its five terminal branches. Entrapment of the radial nerve at the elbow is the third most common compressive neuropathy of the upper limb after carpal tunnel and cubital tunnel syndromes. Because the incidence is relatively low and many agents can compress it along its whole course, entrapment of the radial nerve or its branches can pose a considerable clinical challenge. Several of these agents are related to normal or variant anatomy. The most common of the compressive neuropathies related to the radial nerve is the posterior interosseus nerve syndrome. Appropriate treatment requires familiarity with the anatomical traits influencing the presenting symptoms and the related prognoses. The aim of this study is to describe the compressive neuropathies of the radial nerve, emphasizing the anatomical perspective and highlighting the traps awaiting physicians evaluating these entrapments.

## Introduction

The radial nerve (RN) arises from the posterior cord of the brachial plexus (C5 – Th1). It initially descends from the axilla posterior to the brachial artery and heads between the long and medial heads of the triceps brachii. Next, it takes course in the radial groove towards the lateral side of the arm, simultaneously passing from the posterior to the anterior compartment [1]. Thereafter, it pierces the lateral intermuscular septum (LIMS) and then travels between the brachioradialis and brachialis muscles. At the level of the head of the humerus, it gives off two terminal branches, deep and superficial. The deep branch of the RN is also called the posterior interosseus nerve (PIN). Some authors differentiate between these two nerves. They consider the deep branch of the RN to be the direct branch, the PIN being its continuation after it passes the supinator muscle.

Before the bifurcation into its terminal branches, the RN gives off lateral branches. These are the branch to the glenohumeral joint, the posterior cutaneous nerve of the arm, the inferior lateral cutaneous nerve of the arm, the muscular branches to the arm’s posterior compartment, the vascular branches to the brachial artery and the periosteum, the posterior cutaneous nerve of the forearm, the muscular branches to the lateral compartment of the forearm, and branches to the elbow joint. The RN innervates all the muscles in the posterior compartment of the arm and the forearm. The motor branches coming directly from the RN supply, in sequence: the triceps brachii, the anconeus, the brachioradialis (BM), and the extensor carpi radialis longus [2, 3]. Cho et al. reported that in 65% of upper extremities in their study, the brachialis muscle received double innervation from the musculocutaneous nerve and the RN, while 35% were innervated solely from the RN (Table 1) [4].

**Table 1:** The main parts of the RN and their functions

	MOTOR	SENSORY
MAIN TRUNK OF THE RADIAL NERVE	• Triceps brachii • Anconeus • Brachioradialis • Extensor carpi radialis longus	• Glenohumeral joint
POSTERIOR CUTANEOUS NERVE OF THE ARM	-	• Posterior aspect of the arm
INFERIOR LATERAL CUTANEOUS NERVE OF THE ARM	-	• Lateral lower aspect of the arm
POSTERIOR CUTANEOUS NERVE OF THE FOREARM	-	• Posterior aspect of the forearm
SUPERFICIAL BRANCH OF THE RADIAL NERVE	• (Variably) Extensor carpi radialis brevis	• Posterior and lateral aspects of the hand and the wrist • Posterior, medial, and lateral aspects of the thumb • Posterior, medial, and lateral aspects of the proximal half of the index finger • Posterior and lateral aspects of the proximal half of the middle finger
POSTERIOR INTEROSSEUS NERVE	• Extensor carpi radialis brevis • Supinator • Extensor digitorum • Extensor digiti minimi • Extensor carpi ulnaris • Abductor pollicis longus • Extensor pollicis brevis • Extensor pollicis longus • Extensor indicis	• Ligaments and articulations of the carpal joints • Periosteum of the radius • Interosseus membrane of the forearm • Muscles of the forearm

The PIN is the primary motor branch of the RN; however, it also carries sensory afferent fibers from the wrist (mainly the ligaments and the joints) and the supplied muscles in the forearm, as well as the radius periosteum and the forearm’s interosseous membrane [5]. It initially takes its course in the cubital fossa under the capsule of the elbow joint between the brachialis and the extensor carpi radialis longus muscles. Next, it heads posteriorly, piercing the belly of the supinator muscle and coursing between its deep and superficial heads. It subsequently lies on the radius and the interosseus membrane and enters the wrist, dividing into branches supplying the muscles of the hand [3].

The superficial branch of the radial nerve (SBRN) is a mainly sensory nerve. It initially takes a course anterior to the supinator muscle and lateral to the radial artery. It is covered by the BM from the anterior side. Next, it heads posteriorly, emerging from under the BM to the subcutaneous layer of the posterior forearm. Finally, at the level of the wrist, it splits into a few branches that reach the posterior part of the thumb, the index finger, and the lateral half of the middle finger [3].

At the beginning of its course, the PIN supplies two muscles: the extensor carpi radialis brevis and the supinator. Just after the exiting of the supinator muscle, it splits into two bundles: the superficial and deep motor branches. The superficial branch is responsible for innervating the extensor carpi ulnaris, extensor pollicis brevis, and extensor pollicis longus. The deep branch is usually longer and typically supplies hand extensors such as the extensor digitorum, extensor digiti minimi, and extensor indicis, but also the abductor pollicis longus [3]. If the variant muscle called the extensor medius propius is present, this also receives motor innervation from the PIN [6].

This nerve supply pattern has variants. Some muscles receive motor innervation not from the PIN but directly from the main trunk of the RN. The extensor carpi radialis brevis and extensor carpi radialis longus were supplied by the RN sequentially in 43% and 26.7% of specimens [7]. However, Konjengbam and Elangbam reported that the extensor carpi radialis brevis was innervated by the PIN in 59%, the SBRN in 39%, and the main trunk of the RN in 2% [8].

The RN can establish communicating branches with the sensory nerves of the forearm, the medial and lateral antebrachial cutaneous nerves. It also has connections with the median nerve and its branches. The PIN contributes to the anterior interosseus branch of the median nerve, and the SBRN communicates with the palmar cutaneous branches of the median nerve [3].

The typical motor deficits related to RN paralysis depend on the location of the pathology. The symptoms of PIN impairment include disrupted extension of the metacarpophalangeal joints of the thumb and fingers and the interphalangeal joint of the thumb. This is called “finger drop.” Normal sensation is retained. The result of paralysis of the extensor carpi ulnaris is radial deviation of the wrist during extension. The impairment of wrist extension resulting from RN injury is called “wrist drop.” It is a symptom of weakening of the extensor carpi radialis longus and extensor carpi brevis. High radial nerve entrapment (HRNE), which is related to neuropathy of the higher levels of the RN, above the radial groove, can involve the triceps brachii and the anconeus. It subsequently leads to impairment of elbow extension. Long-term paralysis of the supplied muscles eventually leads to their atrophy [9].

The RN can be compressed at multiple locations from its origin at the brachial plexus to the ends of its terminal branches. Knowledge of the anatomy of these entrapments is crucial for establishing a correct diagnosis and implementing appropriate treatment (Table 2). Most RN neuropathies are related to its main trunk in the arm. However, the abundance of compressing factors and mimicry of the symptoms of other pathologies can present a real clinical challenge. The compressive neuropathy of the RN with nontraumatic origin is not an ordinary finding; therefore, each patient should undergo complex and thorough examination which may rule out more common causes of symptoms. For this reason, the individual approach to each patient is strongly advised.

**Table 2:** The structures involved in entrapment of the RN and its branches

NERVES	RADIAL NERVE	POSTERIOR INTEROSSEUS NERVE	SUPERFICIAL BRANCH OF THE RADIAL NERVE
COMPRESSING STRUCTURES	• Lateral head of the triceps brachii • The band from the lateral head of the triceps brachii • Lateral intermuscular septum • Brachioradialis • Teres major • Fibrous tissue in the triangular space • Pulsating artery	• The fibrous tissue of the radial head • The leash of Henry • The extensor carpi radialis brevis and its aponeurosis • The arcade of Frohse • The distal end of the supinator muscle • The wrist bones • Extensor indicis • Extensor digitorum brevis manus	• Brachioradialis • Fascial ring of brachioradialis

## High radial nerve entrapment

The term HRNE includes all compressions along the course of the RN from its origin in the brachial plexus to its bifurcation above the cubital fossa. The symptoms include both motor and sensory deficits. The weakness and dysfunction can affect not only the muscles supplied directly by the RN but also by the PIN. Compression located in the proximal parts of the RN can also lead to pain around the scapula, which radiates into the proximity of the radial groove, the supinator muscle, and the radial styloid process [10]. The most common etiology of HRNE is a direct effect of humeral shaft fractures and their distant complications, or is associated with fracture treatment [11]. RN palsy occurs in 2–17% of humeral shaft fracture cases, but such fractures are responsible for 70% of RN neuropathies [12, 13]. The most common locations of nontraumatic RN injuries are the radial groove, elbow, and PIN (respectively 43.5%, 34.8%, 17.4%) [12]. The incidence of post-fracture, iatrogenic RN palsy (in patients without RN injury resulting from trauma) is estimated at 7% [14]. Hugon et al. found that compression in the area between the brachialis and BM was caused by a fibrous scar and an osseus tunnel, byproducts of the fracture and excessive growth of the bony callus [15]. The symptoms of RN neuropathy in this region can also result from a tumor directly compressing the RN [16].

The RN can also be entrapped above the level of the radial groove. Ng et al. described a case of radial nerve palsy originating in the triangular space as a result of severe hypertrophy of the teres major muscle in an élite bodybuilder [17]. Also, Sebastian reported RN compression caused by soft tissue dysfunction manifesting as fibrous bands arising from the triceps brachii and teres major and narrowing the triangular space [10].

The RN is susceptible to iatrogenic palsy especially during management of humeral shaft fractures and distal humeral fractures. The nerve may suffer mainly from traction but in less frequent occasions also from the exposure, direct damage from a drill or an implant, and pressure from a retractor. According to Hara et al., 94% of iatrogenic injuries of the peripheral nerves is a result of the intraoperative surgical damage [18]. The other cases were caused by the post-surgical inflammatory response leading to fascicular constriction of the nerve. Claessen et al. stated that transient dysfunction of the RN occur in 20% patient treated with the lateral exposure of the humerus, 11% with posterior exposure, and 4% with anterolateral exposure [14].

HRNE needs to be differentiated carefully from cervical radiculopathy which is the compression of the cervical nerve roots. The typical symptoms consist of unilateral pain which originates in the neck and radiates to the regions supplied by the affected nerve. The other symptoms include sensory loss and muscular weakness along the corresponding dermatome or myotome. However, lack of symptoms outside the neck does not exclude the radiculopathy. Electromyography is considered useful in differentiation with the peripheral nerve neuropathies but in order to avoid the false-positive or false-negative results, it is advised to use it corelated with the clinical context and with the assist of imaging such as radiographs, MRI, or computed tomography [19, 20]. This evaluation is helped by performing Wainner’s cluster, comprising four tests: Spurling’s test, ipsilateral neck rotation, distraction, and the upper limb tension test. When the three of the four tests are positive, radiculopathy can be confirmed [10].

RN entrapments do not necessarily have to result from focal or continuous deformations, but can be limited to a lesion in one location. Yamamoto et al. described a case of HRNE compression at the radial groove with no external abnormalities [21]. The main trunk of the RN had four points of constriction, which severely impaired its motor functions. This demonstrated the importance of careful dissection, which helps to ensure that no entrapment points are overlooked. In this case, unintuitively, the patient had no sensory losses; this emphasizes the importance of imaging and electrodiagnostic studies. Yongwei et al. recorded two to five constrictions on one nerve [22].

### Saturday night palsy

“Saturday night palsy” denotes compression of the RN in the arm as a result of direct pressure from a firm object. It is typically depicted as an alcohol-intoxicated person whose arm is hanging over the back of the chair. If prolonged, this position causes symptoms typical of RN compression. The exact location in most cases is the radial groove. In the literature, the term “Saturday night palsy” is often used for all cases of HRNE. The symptoms range from weakness and disruption of precise finger movements to complete paralysis of the muscles supplied by the RN. There is a similar pattern in another familiar disorder called “Honeymoon palsy.” In this condition, the compressing factor is another person sleeping on the patient’s arm [23, 24].

Because the RN follows a superficial course at the radial groove, the most reliable diagnostic option is ultrasonography (USG), which gives high-quality images of structural lesions. The other advantage of this method is the short time required to perform the examination, which is important in acute RN palsy cases [25].

The suspected etiology in some HRNE cases is the so-called punched nerve syndrome. It is described as nerve impairment due to repetitive pulsatile movements of the nearby artery, and occurs under specific anatomical conditions and with particular activities (such as repetitive movements) when the nerve cannot move away and is compressed. This pathology can be visualized using high-resolution USG. The direct effect of pulsatile movements of the vessels on the nerve could be seen. The affected part of the nerve was enlarged and there was a hypoechoic change in the echotexture [26]. Faissner et al. provided evidence that the RN can also be involved in this kind of disorder. They reported it to be the main factor in a case of Saturday night palsy [27].

Treatment of these disorders is usually conservative, as recovery is typically complete after 2 or 3 months. In more severe cases, full recovery can take up to 6 months, but even for these patients the prognoses are good [23]

### Brachioradialis

Compression by the BM is not common, and the few cases vary significantly in their morphological traits. Lee et al. reported a case of a manual worker who presented with weakness in the upper extremity and lack of ability to extend the wrist and fingers, but he suffered no pain [28]. The initial diagnosis of compression involving the triceps brachii was changed intraoperatively as the RN proved to be compressed as it passed between the brachialis and brachioradialis muscles. Postoperative revision of the MRI revealed visible flattening of the nerve at the compression site. This demonstrates that MRI can be reliable diagnostic tool; however, knowledge of the more unusual points of entrapment is required, as they can easily be missed.

Cherchel et al. reported that the BM alone can cause entrapment, with no brachialis muscle involvement [29]. The compressive force was against the humerus and the nerve was covered by aponeurotic tissue. It was demonstrated that the point of excessive pressure can be provoked by muscular tissue at the humeral origin of the BM. The RN was squeezed as it entered the space between the brachialis and the BM. The subject in this case was again a manual worker.

The possibility of RN entrapment should also be considered when accessory muscles are present. Mehta et al. found an anomalous muscle originating from the distal end of the deltoid muscles and merging with the BM at the level of the lateral epicondyle of the humerus [30]. The brachialis and the BM were also fused together at the lateral epicondyle. This can also be considered an accessory head of the BM, not a separate entity. The RN passed tightly between the accessory and the main brachioradialis muscles. This was therefore considered to be the potential site of the compression, especially when there was extensive physical effort.

### Lateral intermuscular septum

The LIMS takes its origin at the crest of the greater tuberosity of the humerus and spans to the lateral epicondyle of the humerus, inserting into the annular ligament of the radial head. It separates the arm into anterior and posterior compartments. Because the RN is in direct contact with the LIMS as it descends along the arm, the LIMS can be considered a potential source of entrapment [31]. Compression caused by the LIMS can be either atraumatic or can follow a history of trauma. Chesser and Leslie reported RN entrapment in a patient who suffered RN palsy 3 months after a fracture of the humerus [32]. In such situations, the onset of symptoms can be either immediate or delayed by months or even years after the incident. Bowman et al. described patients who suffered mid-shaft humerus fractures that resulted several years later in chronic anterolateral pain in the arm [31]. There were no motor deficits or pathological sensations. During surgery, compression of the RN was observed at its entrance to the LIMS, with no abnormal tissue in the neighborhood.

The patients in a series of four cases by Adolfsson and Nettelblad did not develop a distinct fibrous arcade directly compressing the nerve [33]. However, the LIMS covered the RN, creating a tunnel along its course. The LIMS was thick, but no nerve abnormalities were noticed. The proposed explanation for the entrapment was local swelling exacerbated by muscular activity and reduced by the release procedure, which either reduced the traction induced by the canal or allowed the triceps brachii to move more posteriorly, weakening its influence on the RN. However, surgery candidates must be carefully selected, as the radical procedure does not always guarantee relief of symptoms in this type of pathology.

### Triceps brachii

This is one of the muscles that can be involved in entrapment of the RN in the arm before it bifurcates into its terminal branches. Manske reported a case of permanent RN palsy caused by the lateral head of the triceps brachii [34]. The RN can also be compressed by repeated injection of analgesics and subsequent myofibrosis. The affected muscles have a “wooden” consistency and visible atrophy with impairment of motion [35]. The symptoms can develop gradually and are not always induced by strenuous activity. Prabhu et al. described an anomalous muscular fascicle spanning between the lateral and long heads of the triceps brachii, passing over the radial groove and covering the RN and the radial artery [36]. This was proposed as a potential site of compression in view of the proximity of the nerve to these structures.

In series of cases presented by Lotem et al., the symptoms of ongoing compression began shortly after strenuous physical effort including exerted extension [37]. All the subjects had muscular body types. In each case, there was spontaneous recovery and complete relief of the symptoms with no surgical intervention. The proposed explanation was a tightened fibrous arch from the lateral head of the triceps brachii below the branches from the RN. The arch subsequently passed the radial groove and inserted below its lateral part into the humerus. The onset of symptoms need not be rapid, as demonstrated by Nakamichi and Tachibana; it took years for their patient to develop serious symptoms [38]. However, even surgical treatment in such chronic cases cannot guarantee complete nerve recovery because the fibrotic changes progress. Jenkins et al. found a type of fibrous or muscular band from the lateral head of the triceps brachii in 75% of the cadaveric upper limbs they studied [39]. They also encountered a specimen with a duplicated band, which was particularly taut during elbow extension with the forearm supinated. Although compression involving the triceps brachii is not common, it should be considered in the differential diagnosis when other explanations have been ruled out.

## Posterior interosseous nerve syndrome

PIN syndrome is considered the most common compressive neuropathy affecting the RN and the third most common neuropathy linked to the main branches of the brachial plexus (after carpal tunnel and cubital tunnel syndromes). However, it cannot be considered common in the general population. Weitbrecht and Navickine found only 12 patients (1%) with radial tunnel syndrome (RTS) among 1051 with confirmed forearm entrapment syndromes [40]. RTS coincided with carpal tunnel syndrome in five patients and with cubital tunnel syndrome in two. Latinovic et al. estimated the incidence of RN entrapment as 2.97 among men and 1.42 among women per 100,000 persons/year [41]. The surgery ratio was 0.5 for men and 0.8 for women per 100,000 person/year. The general tendency is for the incidence to increase up to middle age and subsequently decline. There are differences among studies; Jackson et al. found 3.53 cases per 100,000 person/year [42].

**Fig. 1:**
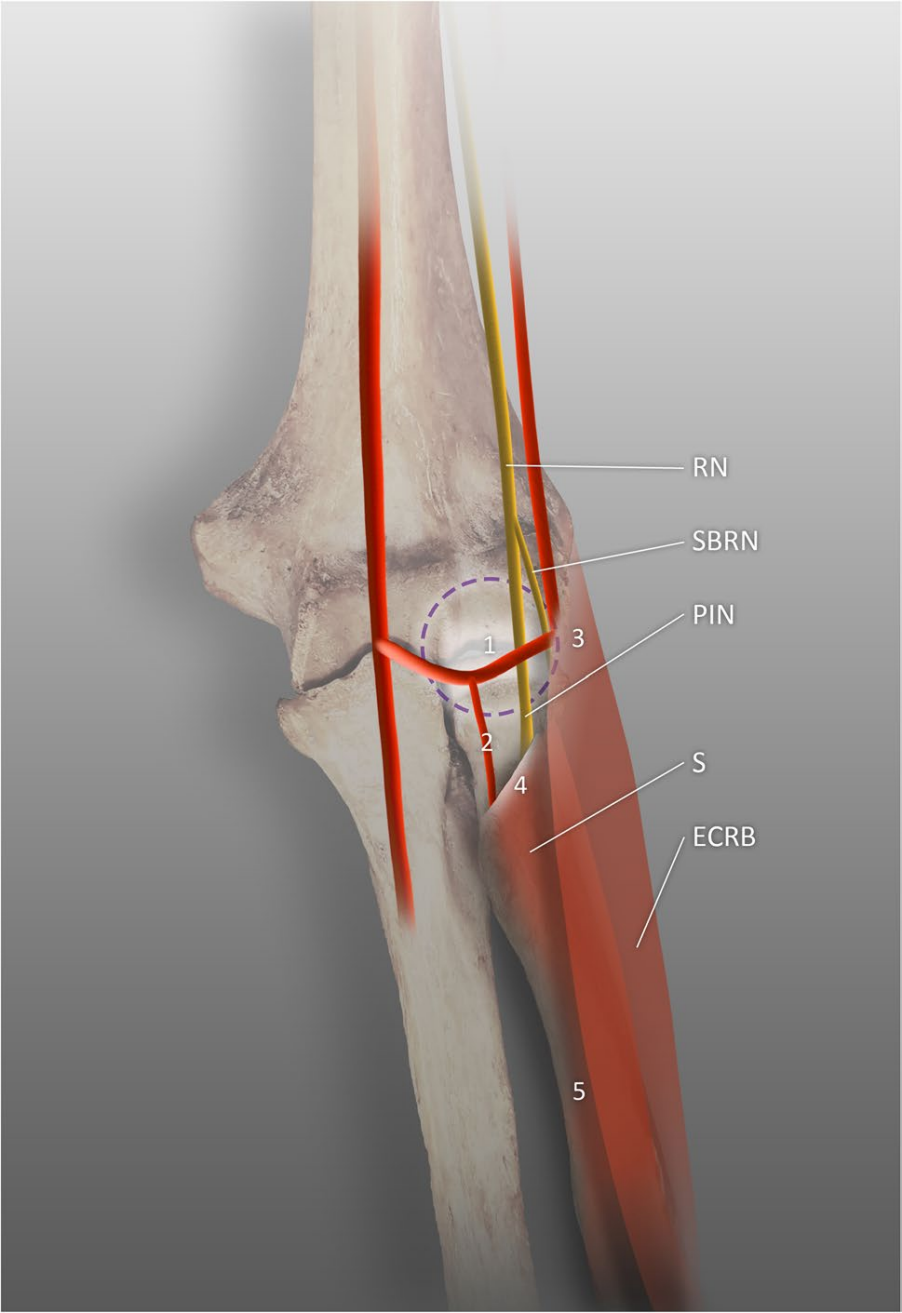
Five basic points of compression of the radial nerve, RN radial nerve, SBRN superficial branch of the radial nerve, PIN posterior interosseous nerve, ECRB extensor carpi radialis brevis muscle, S supinator muscle, 1 fibrous tissue, 2 radial recurrent vessels, 3 margin of the ECRB, 4 proximal margin of the supinator muscle, 5 distal border of the supinator muscle

There are five basic points of PIN entrapment [43, 44]. The most proximal of these is the floor of the radial tunnel, which consists of fibrous tissue arising from the radial head and fusing with the brachialis, BM, extensor carpi radialis brevis, and the superficial head of the supinator. Increased thickness of these bands could be the main reason for entrapment in this sector. The second point is at the level of the radial neck. Here, compression is induced by the recurrent radial vessels, which are often referred as the leash of Henry (LH); their hypertrophy is considered the cause of entrapment. The third point is the thickened, tendinous medial margin of the extensor carpi radialis brevis and the aponeurosis located beneath the muscle, which blends with the deep fascia covering the flexors [45]. The fourth location is the proximal margin of the supinator muscle’s superficial head (more commonly known as the arcade of Frohse (AF) or the supinator arch). The fifth point is the distal border of the supinator. This was reported to be muscular in 65% of cadavers and tendinous in 35% [46]. Gilan et al. found that the distal edge of the supinator was muscular in 57.5%, musculotendinous in 32.5%, and tendinous in 10% (Fig. 1) [47].

The radial tunnel is the space between the radiocapitellar joint and the distal edge of the superficial head of the supinator [44]. Its anterior wall consists of the radial recurrent vessels, BM, and the superficial head of the supinator. The radial tunnel is limited posteriorly by the deep head of the supinator and the capsule of the radiocapitellar joint. Its lateral wall is built from the BM, extensor carpi radialis brevis, and extensor carpi radialis longus. The medial limitations are defined by the tendons of the biceps brachii muscle and the brachialis muscle.

The risk factors identified for workers are high-intensity handgrip force and prolonged exposure of the arms to vibration. In detail, the main work-related risk factors for RN entrapment are as follows: efforts above 1 kg at least 10 times per hour, heavy effort related to a strong grip on objects, and regular working with an extended elbow. Obesity defined as BMI > 30 was not associated with a higher risk of RN compression [48]. Extrinsic compressions of the PIN are rare, although there are reports confirming their existence. Genç et al. recorded the involvement of Canadian crutches. Also, playing instruments can contribute to the development of this disorder [49]. Maffulli and Maffulli showed that violin players suffered from PIN compression with motor and sensory symptoms (including muscular pain) aggravated by movements characteristic of violin playing [50]. The intraoperative finding was a swollen nerve and a fibrotic AF, which suggests that occupational factors can contribute to hypertrophy of this structure.

The AF is considered the most common site of PIN compression [43]. Anania et al. showed that compression occurs at the AF level in 64.4% of cases; 20% of entrapments are in more proximal parts of the nerve and 15.5% distal to the arcade [51]. Ritts et al. found that the AF was the source of compression in 57% of cases, the extensor carpi radialis brevis in 20%, the LH in 13%, and fibrous bands from the radial head in 10% [52]. Fibrous adhesions were found between the PIN and the joint capsule beneath it in 50% of cadavers [8]. Riffaud et al. noted even greater numbers; they found such adhesions in all 25 upper limbs [53].

A histological study by Hill and Hall revealed a possible explanation for a failed surgical treatment of PIN compression despite full release of the nerve [54]. They observed Renaut bodies, which are hyaline structures with fibroblast-like cells and chaotic masses of collagen and elastic fibers. The endoneurium was constricted even after mechanical release since Renaut bodies compromise neural and sometimes vascular function.

**Fig. 2:**
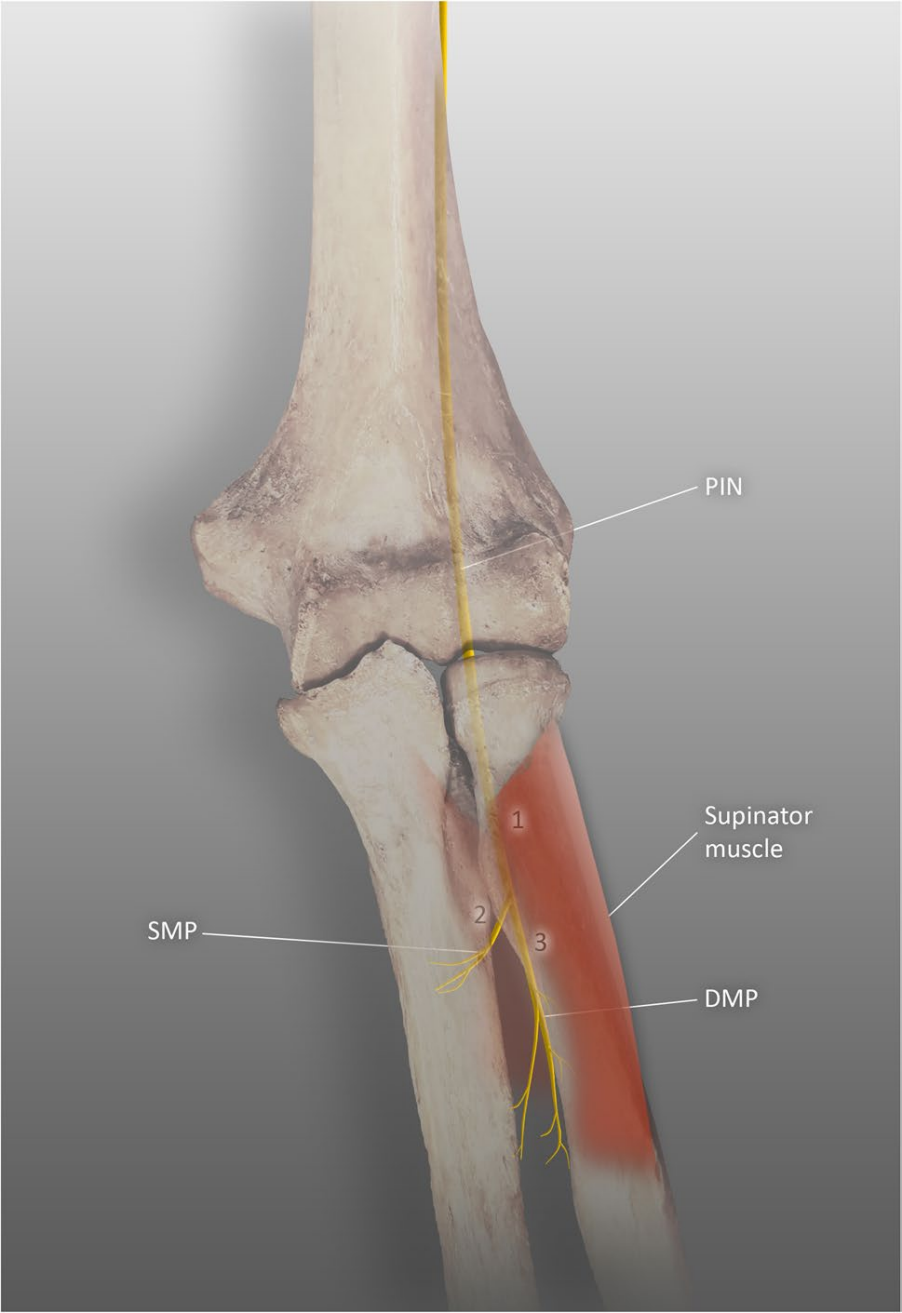
Three types of the proximal posterior interosseous nerve palsy, PIN posterior interosseous nerve, SMP superficial motor branch of the PIN, DMP deep motor branch of the PIN, 1 site of compression in type I, 2 site of compression in type II, 3 site of compression in type III

Three types of proximal PIN palsy can be distinguished according to the location of the compression point [55]. Type I entails simultaneous compression of the superficial and deep motor branches at the entrance to or within the supinator muscle. The symptoms are drop of fingers and thumb. Type II is isolated compression of the superficial motor branch; it affects only the fingers. Its typical location is the exit from the supinator. In type III, the only affected nerve is the deep motor branch; the location is similar to type II (Table 3) (Fig. 2). The visible symptom is drop of the thumb. Pain is not a characteristic symptom of PIN compression, in contrast to RTS.

**Table 3:** Types of PIN entrapment [55]

	TYPE I	TYPE II	TYPE III
AFFECTED BRANCHES	Superficial and deep motor branches	Superficial motor branch	Deep motor branch
LOCATION	The entrance to the supinator muscle and the supinator tunnel	The exit from the supinator muscle	The exit from the supinator muscle
DIGITS AFFECTED	Thumb and fingers	Fingers	Thumb
RECOMMENDED SURGICAL APPROACH	Exposure between the brachioradialis and the brachialis; exploration of the proximal and middle parts of the supinator muscle	Exposure between the extensor carpi radialis brevis and extensor digitorum; exploration of the distal end of the supinator muscle

USG is useful for diagnosing the entrapment syndrome and establishing a possible etiology. The indicators of ongoing pathology are hypoechogenity of the nerve, enlarged nerve fascicles, nerve enlargement, and an increased number of observable blood vessels [44]. In order to preclude false-negative results, Anania et al. suggested moving the USG probe outside the supinator muscle boundaries during examination of the PIN [51].

MRI is irreplaceable in precise visualization of the multiple structures simultaneously as it allows the full coverage of the examined area. The important advantage of MRI compared to USG is its independence from the operator’s skill and experience. It also provides better soft tissue contrast and is capable of depicting the pathological changes within the nerves; however, the main goal of this method is showing the space occupying lesions along the course of the nerve and revealing the denervated muscles [56].

Electrodiagnostic studies serve an important role in assessing the peripheral nervous system as they provide the information about the functioning of the motor and the sensory nerve fibers. They can be used in differentiation and diagnostic process of demyelination and axon loss and are reliable tool in identifying the level of compression [57]. Decreased conduction velocity or reduction of the amplitude in one of the nerve sections might be a sign of its local injury; prolonged latency and decreased velocity are signs of demyelination and reduction in the CMAP amplitude can be linked with axon loss. Electrodiagnostic studies may also be utilized in evaluating the procedure outcomes by comparing pre- and postoperative measurements. The additional tool which helps in assessing the muscle response and nerve conduction is electromyography [57].

However, electrodiagnostic studies require detailed knowledge of the branching of the PIN in the area of the proximal forearm. Furthermore, during the first 2 weeks after occurrence of the palsy, this method can be of limited value and the results can even be negative. After 3 weeks or more, electrodiagnostic studies are reported to be significantly more effective in yielding useful clinical information [58, 59].

When there is pain, swelling, and tenderness along the course of the PIN at two distinct locations, double-level entrapment should be considered. Sponseller and Engber reported a case with the PIN compressed at both the level of the AF and the distal border of the supinator muscle [60]. Also, Yongwei et al. presented three cases of nontraumatic PIN compression with a suspected inflammatory etiology [22]. In four cases described by Kotani et al., all the PIN constrictions were between the division of the supinator motor branch from the main trunk of the nerve and the AF [61]. The characteristic features of more severe lesions were adhesions that induced abnormal nerve rotation. In a case described by Lin et al., there was compression at two locations, the LH and the AF [59]. Casal et al. reported entrapment in the same locations as Lin et al., but there was also a spontaneous rupture of the extensor pollicis longus tendon [62].

The differential diagnosis, apart from ganglia, aneurysm of the radial collateral artery, or traumatic etiologies such as fractures or rupture of the extensors tendons should also consider complications of a systemic disease such as diabetes mellitus, arthritis, multiple sclerosis serum sickness, alcohol abuse, or lead or arsenic intoxication. It can be helpful to identify such factors especially when there is no clear external compressing factor [8, 59, 61].

Surgery is suggested as the primary treatment for PIN palsy only when MRI and sometimes USG have confirmed lesions that directly compress the nerve, or when the symptoms are very severe. In other cases, conservative treatment should be considered the initial option. Since some 30 possible etiologies of PIN compression have been identified, it is difficult to decide a course of treatment suitable for all cases. Therefore, the clinical process should be evaluated individually [63].

Some cases of the PIN palsy may have the iatrogenic origin. The surgical repair of the ruptured biceps tendon can pose a risk of the PIN damage. The palsy may result from compression by a cortical button, a fibrotic hematoma, or direct pressure from the biceps brachii evoked by incorrect positioning of the forearm in the primary surgery. It was reported that the neuropathy also occurred as a result of a direct damage from a drill usage or a dissection along the proximal radius since during supination the nerve is typically located on the shaft of the radius opposite the bicipital tuberosity [64, 65].

### The arcade of Frohse

**Fig. 3:**
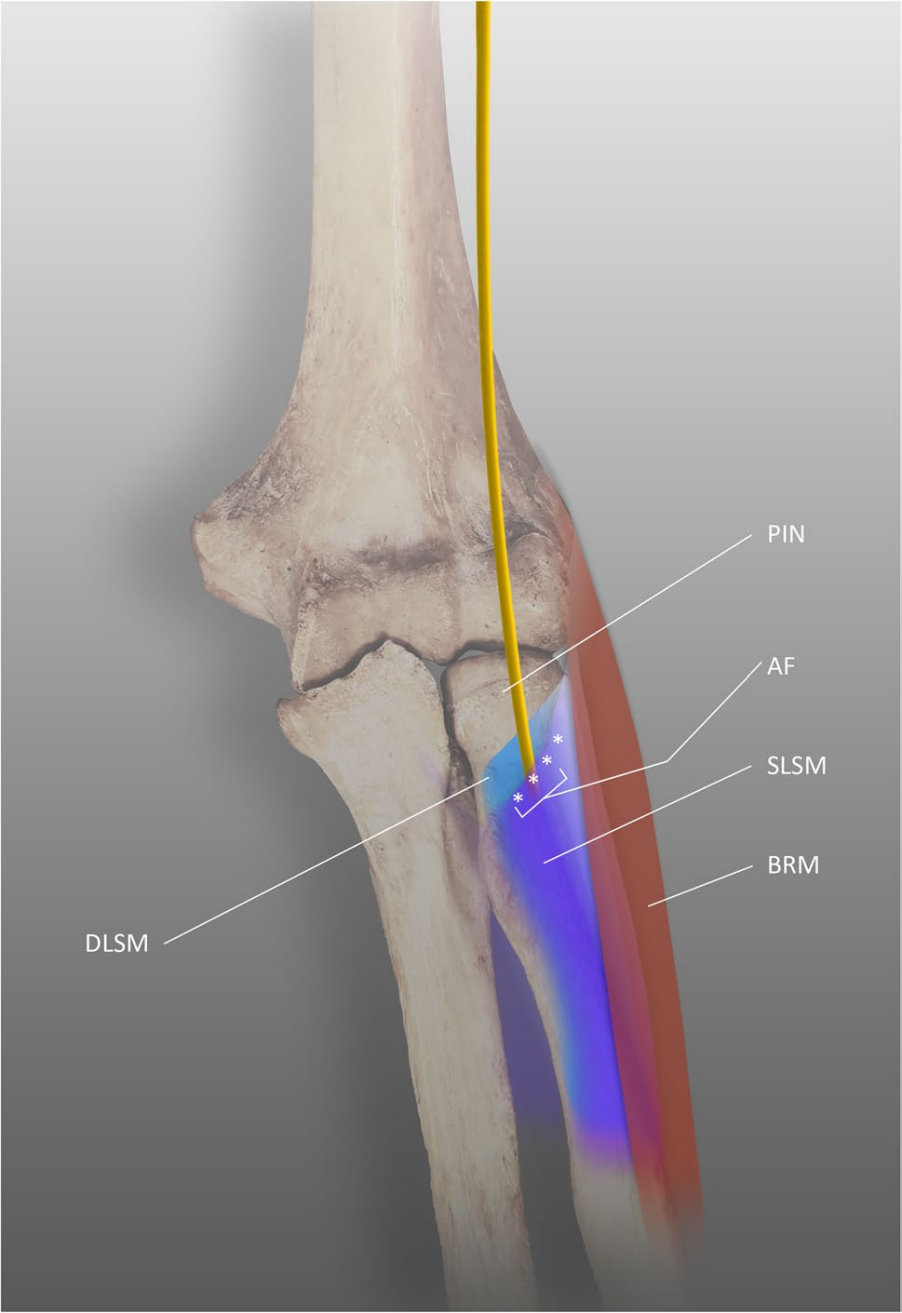
The arcade of Frohse, PIN posterior interosseous nerve, AF arcade of Frohse, DLSM deep layer of the supinator muscle, SLSM superficial layer of the supinator muscle, BRM brachioradialis muscle

The AF is not a homogenous structure; its shape and the type of fibers are variable. Debouck and Rooze proposed a classification of the arcade based on its morphology [66]. Type A indicates a resistant tendinous arcade. Type B is a mixed musculotendinous arch with two types of fiber alternating. Type C is purely muscular and is an extension of the supinator. Type D is a thin, elastic, membranous arcade, with the least coherent structure among the types (Table 4). The AF can adopt three shapes: semicircular in 64%, oblique elongated in 29%, and semioval in 7% of specimens (Fig. 3).

**Table 4:** Characteristics of subtypes of the arcade of Frohse

Variant of the AF	Morphology	Ratio
Type A	The tendinous type	64.10%
Type B	The mixed musculo-tendinous type	21.70
Type C	The muscular type	12.30%
Type D	The membranous type	1.90%

The AF was encountered in 73% of cases. After surgical studies with dominant pathological conditions were excluded, the frequency was counted as 66% [67].

The AF has a broad distribution of measurements in adult specimens. Its mean recorded length is 23.22 mm (maximum 41 mm, minimum 8.6 mm). The width was estimated as 10.13 to 12.8 mm, 11.05 mm on average. The thickness varies from 0.43 to 0.8 mm (mean: 0.67) [67].

“The arcade of Frohse” should be used to denote the tendinous variant of this structure. The proposed systemic name for this particular type of arch is “tendinous arch of supinator muscle” [67]. However, in the literature, the term if often used regardless of the morphological type, a practice followed in the present study.

### The leash of Henry

The LH is a vascular bundle comprising branches from the radial recurrent artery and the surrounding veins. Together, they cross the PIN proximally to the AF. Tubbs et al. found that the LH crossed the PIN in 97% of upper extremities [68]. The LH is typically located from 3.5 to 6 cm below the lateral epicondyle. Superficially, it crosses the head of the radius [68, 69]. A MRI study by Husarik et al. revealed a hypertrophic LH in 15% of subjects, confirmed when a LH consisting of at least six vessels was close to the PIN [70]. The pack of veins crossing the course of the PIN can be also be accompanied by a mass of fibrous tissue, creating adhesive bands, which also contribute to the severity of entrapment [9]. It was proposed that the increased size and number of these vessels can be attributed to manual work, which can generate the stimulus necessary for them to grow [53].

### Distal PIN neuropathies

**Fig. 4:**
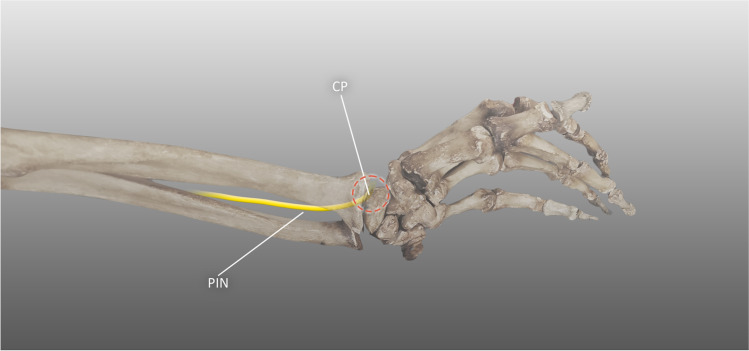
Distal posterior interosseus nerve syndrome, PIN posterior interosseus nerve, CP compression point

The last common site of PIN entrapment is its distal end at the level of the wrist, where the PIN can end in a bulbous expansion just before giving off its final branches. The expansion is probably an adaptation to repetitive dorsiflexion. Enlargement of the nerve causes mechanical impingement between the bones of the wrist during extension, which results in neuropathy syndromes (Fig. 4). The proposed risk factors are excessive enlargement of the nerve and daily activities requiring repetitive movements at the wrist. Conservative treatment is efficient before fibrosis develops; significant scarification and nerve enlargement are indicators for operative treatment [71]. The other causes of PIN irritation at the wrist include trauma to the wrist capsule, postoperative complications, and wrist bones fractures [72].

Some PIN-related abnormalities in the wrist region can be differentiated as completely separate pathologies. The term proposed for chronic posterior wrist pain associated with the fourth compartment is “fourth compartment syndrome.” The fourth compartment is the space in the wrist containing the extensor indicis and extensor digitorum muscles [73]. Direct or indirect compression of the PIN as it passes through the fourth compartment can involve ganglia, the extensor digitorum brevis manus or abnormal extensor indicis muscle, tenosynovitis, or deformations of the carpal bones. The increased pressure inside the compartment squeezes the muscles and involving the PIN. This is considered a cause of the symptoms [73].

The prevalence of the extensor digitorum brevis manus is estimated as 2.5%. It can be palpated as a mass located on the posterior wrist, which can cause pain when the fingers are strenuously extended against resistance or during wrist extension with the palm pushed against a flat surface. In MRI, the muscle can be seen as a homogenous mass along the extensors’ tendons [74]. Typically, the extensor digitorum brevis manus takes its origin at the posterior side of the wrist: at the wrist joint capsule, the distal end of the radius, or the posterior metacarpal surface; or from the radiocarpal ligament. It has from one to four tendons. In the most typical variant, a single tendon reaches the index or the middle finger [75].

## Radial tunnel syndrome

The term often used instead of RTS is “supinator syndrome.” It is also often confused and used interchangeably with posterior interosseus nerve syndrome; both refer to the same nerve and the same compression point [76]. Many authors mentioning RTS actually mean PIN syndrome. However, these disorders should be seen as separate because their clinical presentations are different. The common symptoms of RTS are pain, discomfort, and tenderness over the course of the PIN, which together limit the normal activity of the limb and can also affect the patient at night. The typical sign is worsening of the pain during actions performed by the supinator and extensor carpi radialis muscles. Lister et al. proposed tests such as resisted supination of the extended forearm or resisted extension of the middle finger with the elbow extended [77]. There is no muscle weakness, immobility, sensory deficit, or atrophy in this syndrome.

Whether RTS actually exists is controversial; it has been proposed to be misdiagnosed lateral epicondylitis (more commonly known as tennis elbow), extensor carpi radialis brevis injury, or some other pathology with chronic pain in the lateral part of the forearm [78, 79]. According to Rosenbaum, there is no such thing as a purely sensory radial tunnel syndrome with no concomitant motor deficits caused by PIN compression [80]. He proposed other terms such as “lateral forearm pain syndrome” or “persistent tennis elbow.”

The RTS affects more women than men, typically between their third and fifth decades of life. The symptomatic side is most frequently the dominant hand [81]. The proposed explanation for a purely motor nerve causing sensory deficits is that the PIN also carries unmyelinated C-fibers and small myelinated IIa fibers from the muscles, which are not detectable by electrodiagnostic examination; however, mild compression of them causes the sensations experienced [82].

The symptoms of RTS can mimic lateral epicondylitis, so the two conditions have to be differentiated. The lateral epicondylitis is an overuse injury of the common extensor tendon attaching to the lateral epicondyle of the humerus and should not be associated with the nerve damage. From the clinical point of view, the main difference is the location of the symptoms. The pain related to RTS tends to be located more distally (approximately 3–5 cm) from the lateral epicondyle of the humerus, over the course of the PIN in the radial tunnel; the pain related to lateral epicondylitis is located directly over the lateral epicondyle. Other disorders with similar presentations are direct injury to the RN, pathology of the radiocapitellar joint such as osteoarthritis or synovitis, and muscle lesions [48, 83].

Lateral epicondylitis is sometimes considered to be associated with RTS. The proposed mechanism common to these two conditions is inflammation affecting the extensors and the PIN in its tunnel. However, concurrent surgical treatment for lateral epicondylitis together with PIN release surgery did not yield better outcomes in terms of pain relief or functional improvement [84]. On the other hand, Abhimanyu et al. found that the RN was thickened at the radial groove in 33% of patients and at the cubital fossa in 27% compared to the unaffected side [85]. The only statistically significant clinical outcome was decreased ability when the increased thickness was at the radial groove level. Yet these findings cannot be considered sufficient to link RTS directly to lateral epicondylitis.

MRI findings in RTS, although useful, are not always unambiguous enough to establish the single diagnosis. However, they help significantly in ruling out clinically similar disorders [86]. Ferdinand et al. found denervation edema in 52% and atrophy of the extensors or the supinator muscle [86]. In 28%, there was a mass effect along the course of the PIN. Denervation of the muscles except for diagnostic usefulness could also suggest that the etiology of RTS is in fact related to the PIN, so it is a separate disorder.

No reliable electrodiagnostic findings can help to establish a single diagnosis of RTS because there is no motor denervation. The syndrome is not detectable by pathophysiological or electrodiagnostic tests. There are no observable abnormalities in electromyography or nerve conduction tests in RTS. However, electromyography is considered helpful for ruling out cervical radiculopathy, and detecting denervation or abnormalities in the motor units [82].

**Fig. 5:**
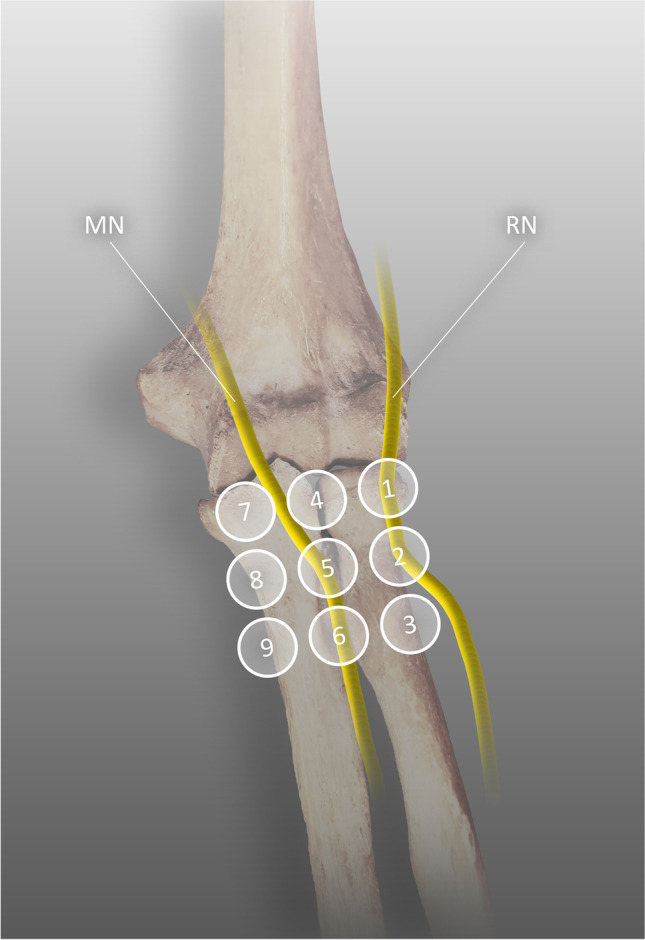
Course of the radial and median nerves in the nine zones, RN radial nerve, MN median nerve

Owing to the difficulties in diagnosing RTS, including inconclusive electrodiagnostic studies and problems with the anatomical aspect of the syndrome, Loh et al. proposed the “rule of nine test” [87]. The proximal surface of the forearm is covered with a square divided into nine equal, smaller squares comprising three columns and three rows. Depending on the position of the radial nerve bifurcation, the PIN in the radial tunnel should be located under the proximal two or the three squares of the lateral column (Fig. 5). In all squares, a tenderness test should be performed to identify the true origin of the symptoms and exclude other closely related pathologies such as lateral epicondylitis.

The outcome of surgical decompression for RTS is unpredictable; 67% of patients who underwent release surgery had good long-term results (minimal or no discomfort), 15% fair (symptoms improved but with moderate pain limiting activities), and 18% poor (pain did not allow normal activities to be resumed). Concomitant compressive neuropathies make the procedure more likely to be unsuccessful. Patients with coexisting entrapments had good results in 57% of cases, fair in 14%, and poor in 29%. There is a similar trend when lateral epicondylitis is present: there was a good outcome in 43% of patients [88]. The concept of concomitant release of the PIN and SBRN was successful 67–92% of surgeries. Interestingly, singular release of the SBRN was also considered successful in most cases [89]. Stanley concluded that complete relief of symptoms after surgery should be expected in less than 9 months [90]. After that time, the surgical treatment should be considered unsuccessful. The initial diagnosis should be reviewed and extended with further explanations such as atypical lateral epicondylitis or double crush syndrome.

## Wartenberg syndrome

Wartenberg syndrome (WS) is a compression neuropathy affecting only the SBRN. Its incidence is estimated as 0.003% in the general population [81].

The SBRN is thought to suffer external compression at its distal end in the area of the wrist and hand because of its subcutaneous course in direct proximity to the radius. Repetitive action of the external force can lead to a fibrous reaction, which results in symptoms of entrapment [91]. Some US prisoners during Operation Desert Storm suffered purely sensory symptoms in the area supplied by branches of the SBRN because of prolonged handcuff wearing [92]. There is also evidence that even too tight a watch strap or wristband can contribute to the nerve disorder [93].

The risk to the SBRN is greatest where it makes a transition from the deep to a subcutaneous course at the posterior border of the BM [76]. The typical symptoms of WS are pain, numbness, and paresthesia in the posterior and dorsal parts of the forearm. These symptoms tend to radiate to the wrist, the index finger, and the thumb [76]. WS is sometimes confused with de Quervain syndrome, but patients with the latter mainly experience pain during movements with the wrist and thumb, while those with WS more often have symptoms unprovoked by any movement [94]. Lanzetta and Foucher reported that compression of the SBRN was associated with de Quervain syndrome in 50% of cases [95]. Long-lasting de Quervain syndrome can result in distention of the extensor compartment and excessive stretching of the SBRN, leading to WS [96]. A physical test helpful for diagnosing WS is Tinel’s sign; however, it is not specific to this disorder.

Anatomical variants of the BM have been reported as agents compressing the SBRN. Spinner and Spinner reported entrapment of the SBRN by an accessory BM [97]. The muscle’s origin was the distal humerus and its point of insertion was the proximal part of the radius. The SBRN passed between the brachialis and the accessory muscle and was compressed at the level of the lateral epicondyle of the humerus. The posterior fascia of the muscle was thickened. Other factors contributing to the neuropathy were the size and course of the muscle and the proximity of the bone.

**Fig. 6:**
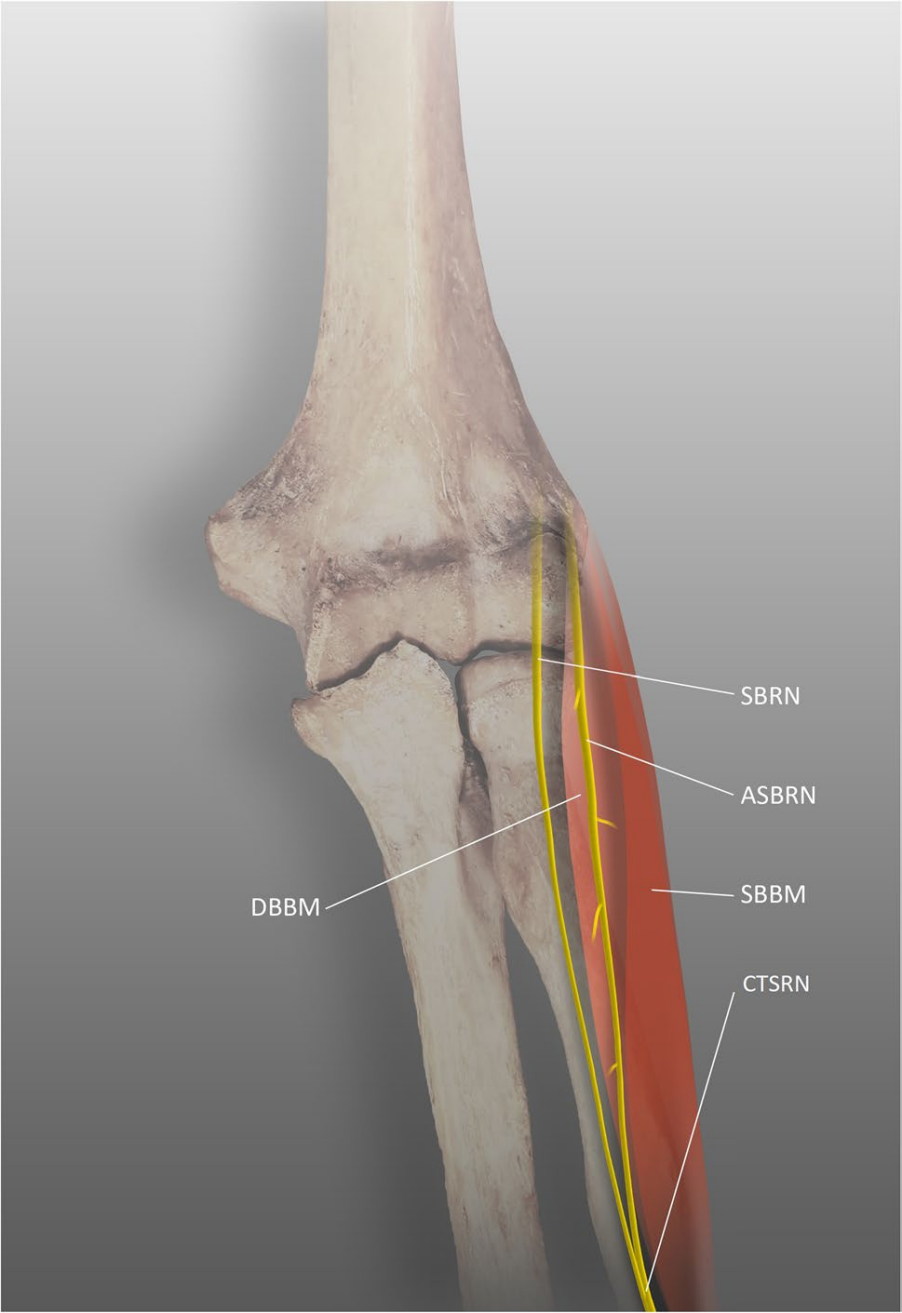
Two-bellied brachioradialis muscle, SBRN superficial branch of the radial nerve, ASBRN accessory superficial branch of the radial nerve, DBBM deep belly of the brachioradialis muscle, SBBM superficial belly of the brachioradialis muscle, CTSRN common trunk of the superficial branch of the radial nerve

Herma et al. presented a constellation of variants: a duplicated SBRN with the two-belly variation of the BM [98]. The RN divided into three branches: the PIN, the SBRN, and the accessory SBRN. The SBRN ran under the deep belly of the BM, and the accessory SBRN coursed under its superficial belly. Along its course, it was crossed by vessels in two separate places and pierced the muscle bundles. The nerves merged after exiting the BM (Fig. 6). It was suggested that because of its course between the layers of the BM and direct penetration into the muscle, the SBRN could potentially be compressed by a hypertrophy. A similar case was reported by Murphy and Blair; a patient with an accessory SBRN suffered from symptoms of its entrapment [99].

The other possible sites of excessive pressure on the nerve are the more distal parts of the upper limb. The symptoms are presumably related to twisting injuries of the forearm or wrist or crushing contusions. Other common contributing factors are activities involving pronation or supination of the forearm. The site of entrapment is where the SBRN exits beneath the deep fascia of the forearm, at the fascial connection between the BM and the extensor carpi radialis longus muscle [100]. This area, together with the further subcutaneous course of the SBRN on the posterior border of the BM, is considered to pose the greatest risk for nerve compression [76]. The proposed explanation is that scar tissue at the wrist resulting from former injuries and anatomical conditions in the forearm, combined with certain patterns of movement, contribute to increasing the friction on the SBRN [100].

The SBRN was also reported to be entrapped by a fascial ring at the posterior edge of the BM, which embraced the nerve as it pierced the muscle’s tendon [101].

A BM with split tendons was discovered in 6% of the cadavers examined. In 55% of those (3.3% of the total), the SBRN passed between these tendons at their bifurcation [102]. Dhuria et al. described a SBRN emerging to the subcutaneous layer between two muscle slips of the BM, while Turkof et al. observed a similar situation but the SBRN was located between the tendons of this muscle [103, 104]. The same mechanism was proposed in both these situations: the nerve was pinched between two parts of the muscle during repetitive pronation of the forearm with the wrist in the ulnar deviation.

A detailed interview including history of former traumata is also important in cases with suspected WS. The possibility of a bone fragment compressing the SBRN as a result of a Colles fracture is rare but should not be excluded, especially in the distal parts of the forearm where the nerves and bone are in immediate proximity [105].

Besides pathologies with anatomical or traumatic etiologies, the SBRN can also be compressed by excessive tissue growth. Ganglia constitute 50–70% of all soft tissue tumors in the hand; 60–70% of these arise from the posterior wrist. Their presence in the anatomical snuffbox caused compression [106]. However, they can also be encountered in more proximal parts of the nerve such as at the elbow [107]. The symptoms can be caused by any kind of tumor close to the nerve, from relatively common ones such as fibromas [108] to the least expected such as dilated lymphatic vessels [109]. Mackinnon and Dellon suggested that because the lateral antebrachial cutaneous nerve and the SBRN overlap partially or completely in the posteroradial part of the wrist in 75% of cases, an injury related to the SBRN (such as a neuroma) can be much harder to treat than anywhere else, as the other nerve can be a concomitant, but avoided, source of the pain [110].

The subcutaneous course of the SBRN makes it prone to iatrogenic injuries during surgeries. External fixator pins used in management of the distal radius fractures are usually located in the proximity of the nerve as it travels on the distal radial side of the forearm [111]. Insertion and removal of the Kirschner wire are associated with potential damage of the SBRN both during the procedure and as an effect of the excessive compressive growth of scar tissue [112]. The branches of the SBRN near the extensor retinaculum may also suffer in first dorsal wrist compartment surgeries and during ganglia removal [111, 113]. The particular attention must be taken during the harvesting the radial artery for coronary artery bypass surgery as the vessel is closely associated with the nerve at the level of the wrist in 72% of cases [114].

Although electrodiagnostic studies can help, they are not always sufficient to confirm WS. Lanzetta and Foucher reported that 50% of the patients examined had no detectable nerve conduction abnormalities, so further evaluation was necessary [95].

## Conclusions

Compressive neuropathies of the RN can present a clinical challenge. Although they are rare, there is a wide range of possible etiologies and regions of the nerve where compression can occur. The RN compression syndromes need to be differentiated both with the systemic diseases and the local pathologies such as the lateral epicondylitis or the cervical radiculopathy as they may exhibit similar symptoms. Even though most of the RN injuries have traumatic etiology, the nontraumatic RN injuries represent a significant portion of the neuropathies, thus they cannot be neglected in the diagnostic process and each patient should be evaluated individually. Knowledge of the anatomical variations and contributing factors is important for solving clinical problems.

## Data Availability

Please contact authors for data requests (Łukasz Olewnik PhD—email address: lukasz.olewnik@umed.lodz.pl).
